# *Taenia solium* Cysticercosis, Irian Jaya, Indonesia

**DOI:** 10.3201/eid0907.020709

**Published:** 2003-07

**Authors:** Toni Wandra, Akira Ito, Hiroshi Yamasaki, Thomas Suroso, Sri S. Margono

**Affiliations:** * Ministry of Health, Jakarta, Indonesia; † Asahikawa Medical College, Asahikawa, Japan; ‡ University of Indonesia, Jakarta, Indonesia

**To the Editor:** Cysticercosis, a tissue infection caused by accidental ingestion of eggs released from humans harboring the pork tapeworm, *Taenia solium* (TsCysti), is one of the most serious reemerging parasitic diseases worldwide (*1*). Taeniasis is an intestinal infection caused by the adult stage of the large tapeworm. Carriers of *T. solium* acquire infection through eating undercooked pork contaminated with cysticerci (larvae). Although most Indonesian people are Muslim and do not eat pork, infection with *T. solium* has occurred in some areas or islands where most local people are Christian or Hindi.

The area most affected by this infection is Irian Jaya, Indonesia, the western half of New Guinea Island (*2*–*4*). In field surveys conducted in 2000 and 2001, we found that 5 (8.6%) of 58 local people and 7 (11%) of 64 local dogs living approximately 1 km from the local capital city, Wamena, in Jayawijaya District, harbored adult tapeworms and cysticerci of *T. solium*, respectively (*5*,*6*). We have further seroepidemiologic data from 1996 and molecular confirmation of subcutaneous nodules (SCN) as cysticerci of the *T. solium* Asian genotype. We believe this organism is an emergent problem in Irian Jaya.

We previously reported that TsCysti was highly endemic in Jayawijaya District, Irian Jaya (*2*–*6*). A total of 96 local people >18 years of age from Assologaima, Jayawijaya District, were chosen at random and examined by serologic testing and by administering questionnaires in February 1996 after the local and Indonesian governments gave their ethical approval. The 96 persons were divided into three groups on the basis of a history of epileptic seizures (ES, n=17), physical examination of SCN by palpation (n=32), or good health (including no ES or SCN; n=47). A total of 14 subcutaneous nodules removed from 14 men in both ES and SCN groups were confirmed to be cysticerci of *T. solium* by morphologic observation and to be *T. solium* Asian genotype by mitochondrial DNA analysis with cytochrome *c* oxidase subunit 1 gene (*3*,*7*). For serologic analysis, we conducted an enzyme-linked immunosorbent assay (ELISA) that used glycoproteins purified from cyst fluid of *T. solium* cysticerci by preparative isoelectric focusing (fractions of pH 9.1) (*8*) in 2001.

On the basis of serologic results, 12 (70.6%) of 17, 20 (62.5%) of 32, and 12 (25.5%) of 47 of ES, SCN, and healthy groups, respectively, were infected with the larval stage of *T. solium*. Serologically positive rates increased to 83.3% (10/12) of people with subcutaneous nodules in the ES group. A follow-up study of seropositive persons in the healthy group in 1997 showed that five of eight persons had ES (two persons), headache (one person), or SCNs in upper arm (two persons). Seropositive persons in all three groups (ES, SCN, and health) were considered to be infected with TsCysti. Persons of the SCN and healthy groups who showed optical density values higher than the cut-off value were considered to have asymptomatic TsCysti cases.

The local persons we examined ranged from 18 to 29 years of age (n=30), 30–44 years of age (n=36), and >45 years of age (n=30). Seropositive persons (n=12) from the ES group (n=17) were 18 to 29 years of age (40.0%, 2/5), 30–44 years (71.4%, 5/7), and >45 years (100%, 5/5). The prevalence of TsCysti did not vary statistically by sex (males 53.6% [37/69] versus females 33.3% [9/27], Pearson’s chi-square test, p=0.074).

That 14 persons confirmed to have subcutaneous cysticerci of *T. solium* were seropositive strongly suggests that the serologic test (ELISA) is highly reliable for detecting TsCysti in patients, whether their infection is symptomatic or asymptomatic. In contrast, one of the following scenarios was expected for cases in three of five persons in the ES group who did not have SCN and were seronegative: 1) the case was not due to TsCysti, 2) the case was caused by TsCysti but without antibody response, rather common in cases of a solitary cyst, or 3) the case was caused by TsCysti with calcified cysts and without antibody response. Twelve (approximately 40%) of seronegative persons from the SCN group (n=32) were expected to have cases of TsCysti without antibody response or to have calcified cysts without antibody response. Cases without antibody response would be most expected because of the heavily contaminated environment in Papua (*3*–*6*). However, further evaluation with computed tomography or magnetic resonance imaging scans is necessary. Based on serologic results and mitochondrial DNA confirmation of *T. solium* Asian genotype (*3*,*7*), we concluded that 47.9% (46/96) of local people examined at random, 53.6% of men (37/69) and 33.3% of women (9/27) >18 years of age had TsCysti.

An additional 30 local people in non–TsCysti-endemic Merauke District underwent serologic testing. One woman had an exceptionally high antibody titer. She was a transmigrant from another island (South Sulawesi Province). Although Paniai, Jayawijaya, and Manokwari Districts are contaminated with *T. solium* taeniasis and cysticercosis (*2*–*4*), no additional critical evidence exists to show that Merauke District has already been contaminated with this parasite.

Taeniasis and cysticercosis may have been accidentally introduced into Irian Jaya in 1969 when the country was governed by Indonesia, since the governing body came from Bali, the only area in Indonesia where TsCysti was exclusively endemic (*2*). The contaminated areas in Irian Jaya have increased from the central area (Paniai), to the east (Jayawijaya) (*3*), and then to the west (Manokwari), where 54 TsCysti cases have been reported (Papua Province Health Office Services, 1997, unpub. data). We wanted to know if taeniasis/cysticercosis had been introduced into the eastern half of New Guinea Island, called Papua New Guinea (PNG) (*9*). We had already serologically confirmed that 16 (3.0%) of 541 local residents and Irianese refugees in Alice River villages along the border in PNG had asymptomatic TsCysti (Ito et al., unpub. data). Follow-up surveys will be crucial in several other districts including Merauke District in Irian Jaya, PNG, and other islands such as Timor Island, where most of the population is Christian and many suspected cases have recently been reported by the District Health Office Services (*10*). Schoolchildren should also be checked so that cases can be detected and treated early. Sustainable education of the local community in Papua, Indonesia, and Papua New Guinea is also necessary.
